# *In Vitro* Antileishmanial Activity of Essential Oil of *Vanillosmopsis arborea* (Asteraceae) Baker

**DOI:** 10.1155/2013/727042

**Published:** 2013-07-09

**Authors:** Aracélio Viana Colares, Fernando Almeida-Souza, Noemi Nosomi Taniwaki, Celeste da Silva Freitas Souza, José Galberto Martins da Costa, Kátia da Silva Calabrese, Ana Lúcia Abreu-Silva

**Affiliations:** ^1^Universidade Federal do Maranhão (UFMA), 65080-805 São Luís, MA, Brazil; ^2^Rede Nordeste de Biotecnologia (RENORBIO), 65080-805 São Luís, MA, Brazil; ^3^Faculdade Leão Sampaio (FALS), 63180-000 Juazeiro do Norte, CE, Brazil; ^4^Laboratório de Imunomodulação e Protozoologia, Instituto Oswaldo Cruz (FIOCRUZ), 21040-900 Rio de Janeiro, RJ, Brazil; ^5^Unidade de Microscopia Eletrônica, Instituto Adolf Lutz (IAL), 01246-000 São Paulo, SP, Brazil; ^6^Laboratório de Pesquisa de Produtos Naturais, Universidade Regional do Cariri (URCA), 63105-000 Crato, CE, Brazil; ^7^Departamento de Patologia, Universidade Estadual do Maranhão (UEMA), 65055-310 São Luís, MA, Brazil

## Abstract

The search for new immunopharmacological chemical agents to treat various diseases caused by bacteria, fungi, and protozoa, such as leishmaniasis, for example, has led to the exploration of potential products from plant species and their main active ingredients. Antimonial drugs are the current treatment for leishmaniasis. These drugs cause major side effects and frequent discontinuation of treatment. In this study, we evaluated the *in vitro* leishmanicidal activity of essential oil of *Vanillosmopsis arborea* (VAEO) and its major compound **α**-bisabolol against *Leishmania amazonensis*. The essential oil and **α**-bisabolol showed activity against promastigotes (IC_50_ 7.35 and 4.95 **μ**g/mL resp.) and intracellular amastigotes (IC_50_ 12.58 and 10.70 **μ**g/mL, resp.). Neither product showed any cytotoxicity on treated macrophages. The ultrastructural analysis of promastigotes incubated with VAEO or **α**-bisabolol at 30 **μ**g/mL, showed morphological changes with the accumulation of vesicles electrodense lipid inclusions. The results give evidence that both VAEO and **α**-bisabolol have potential as new therapeutic agents against leishmaniasis.

## 1. Introduction

Natural products obtained from plants have been used in the treatment of infectious diseases, especially in developing countries. It is estimated that about 25% of medicines are derived directly or indirectly from herbal products [1]. Several studies have shown that various plant species have inhibitory action against certain types of parasites such as *Leishmania amazonensis* [2], *Leishmania chagasi* [3], and *Leishmania infantum* [4].

Leishmaniasis is a chronic disease caused by protozoan parasites belonging to the genus *Leishmania*. It is an endemic disease that constitutes a serious public health problem, threatening about 350 million people in more than 90 countries [5]. *Leishmania* species have a wide distribution among tropical and subtropical countries, including several countries in Latin America, Africa, India, part of Western Asia, and some Central European countries bordering the Mediterranean. The disease presents two clinical forms: cutaneous and visceral and the former can be subdivided into localized cutaneous leishmaniasis, diffuse cutaneous and mucocutaneous [6]. The cutaneous form is the most common, affecting about 1 to 1.5 million people per year [7, 8]. In Brazil, cutaneous leishmaniasis is present in almost all states of the federation, and *L. amazonensis* is the main etiologic agent [7].

Drugs such as sodium stibogluconate (Pentostam), amphotericin B, pentamidine, and antimony N-methylglucamine (Glucantime) are used for leishmaniasis treatment. The pentavalent antimonials have been the mainstay of treatment for human leishmaniasis in recent decades, with cure rates ranging from 60 to 90% [9, 10]. However, treatment with pentavalent antimonial is generally long expensive and toxic and this prolonged course of treatment has caused the development of resistance to these drugs [11, 12].

Thus the development of antileishmanial agents from natural products has become important to find an alternative drug with low toxicity and high efficacy [13].

Essential oils (EOs) are natural compounds and complex mixtures obtained from different parts of plants such as the flowers, leaves, bark, stems, wood, roots, fruits, or seeds and are commonly used in folk medicine to treat various types of diseases [14, 15]. Most of the EOs are comprised of terpenes, especially monoterpenes and sesquiterpenes. Several studies by different research groups have shown biological activity of EOs and their major compounds against protozoal diseases such as leishmaniasis. For example, the essential oil of *Chenopodium ambrosioides* [16], *Cymbopogon citratus* [17] that both contain citral, have been evaluated for their antileishmanial properties. Also the studies of Escobar et al. [18] who tested 19 essential oils, five of which were from different species of *Lippia* and all of them were evaluated for their antileishmanial properties.

*Vanillosmopsis arborea* Baker is native to the Araripe National Forest, in the Northeast of Brazil in the state of Ceará [19]. There are few studies concerning the traditional use of this plant. However, biological and pharmacological studies have shown that the essential oil of *V. arborea* (VAEO) presents antimicrobial [14], anti-inflammatory [20], and gastroprotective activity [21]. The present study characterized the essential oil of *Vanillosmopsis arborea* chemically and evaluated its leishmanicidal and cytotoxic activity. Its major compound *α*-bisabolol was also evaluated.

## 2. Materials and Methods

### 2.1. Plant Material

*Vanillosmopsis arborea* Baker (Asteraceae) was collected in Crato County, Ceará State, Brazil. The plant material was identified by Dr. Arlene Pessoa, and a voucher specimen was deposited under number 9493 at the Herbarium “Dárdano de Andrade Lima” of the Universidade Regional do Cariri (URCA).

### 2.2. Essential Oil Extraction

The oil was extracted using a Clevenger-type apparatus. The analysis of the volatile constituents was carried out in a Hewlett-Packard GC/MS, model 5971. The stems were dried (850 g), according to (traditional) folk medicine, and reduced in size, and EO extraction was carried out by hydrodistillation for 2 h using a Clevenger-type apparatus, resulting in an essential oil yield of 0.4% (3.40 mL). After the oil was collected, it was dried using anhydrous sodium sulfate and subsequently stored under low light conditions at 10°C until analysis [22].

### 2.3. Essential Oil Analysis

Oil analysis was performed using a Shimadzu GC MS-QP2010 series (GC/MS system) under the following conditions: Rtx-5MS capillary column (30 m × 0.25 mm, 0.25 *μ*m film thickness); carrier gas—helium at 1.5 mL/min; injector temperature—250°C; detector temperature—290°C; column temperature—60°C–180°C at 5°C/min, then 180°–280°C at 10°C/min (10 min); scanning speed—0.5 scan/sec from *m*/*z* 40 to 350; split ratio—1 : 200; injected volume—1 *μ*L of [25 *μ*L (essential oil)/5 mL CHCl_3_] (1 : 200); solvent cut time—2.5 min; and mass spectrometer—operated at 70 eV of ionization energy. The identification of individual components was based on their mass spectral fragmentation according to the mass spectral library NIST 08, retention indices, and comparison with published data. 

### 2.4. Parasites

Promastigote forms of *Leishmania amazonensis* (MHOM/BR/76/MA-76) were cultured at 26°C in LIBHIT media supplemented with 10% fetal bovine sera (Gibco, USA) and 100 U/mL of penicillin (Gibco, USA). Cultures were used only up to a maximum of ten *in vitro* passages [23].

### 2.5. Animals

Female BALB/c mice 4–6 weeks old were purchased from Centro de Criação de Animais de Laboratório, Instituto Oswaldo Cruz, Rio de Janeiro, and maintained under pathogen-free conditions. The animals were handled in accordance with Guidelines for Animal Experimentation of the Colégio Brasileiro de Experimentação Animal. The local Ethics Committee on Animal Care and Utilization approved all procedures involving the animals (CEUA FIOCRUZ-LW72/12).

### 2.6. Cell Cultures

The macrophage J774.G8 line was cultured in RPMI 1640 medium (Sigma, USA) supplemented with 10% fetal bovine sera, penicillin (100 U/mL), and streptomycin (100 *μ*g/mL) at 37°C and 5% CO_2_. Female BALB/c mice were inoculated with 1 mL of sodium thioglycolate 3%, and after 72 hours the peritoneal macrophages were harvested with PBS solution. The harvest was centrifuged at 4000 rpm, and the cells were suspended in RPMI medium supplemented as described before and cultured at 37°C and 5% CO_2_.

### 2.7. Activity against Promastigote Forms

Promastigote forms of *L. amazonensis* (10^6^ parasites/mL) from a 2–4-day-old culture were placed in 96-well plates in the presence of different concentrations of VAEO and *α*-bisabolol (Sigma-Aldrich, ≥95% (GC)) (1.9–60 *μ*g/mL, for both products) to a final volume of 200 *μ*L per well for 24 hours. Wells without parasites were used as blank, and wells with only parasites were used as control. After the treatment, the viability of parasites was evaluated by the tetrazolium-dye (MTT) colorimetric method modified by Mosmann [24]. MTT (5 mg/mL), a volume equal to 10% of the total, was added to each well. After 2 hours, the plate was centrifuged at 4000 rpm; then 150 *μ*L supernatant was removed from each well and 100 *μ*L of DMSO were added to dissolve the formazan. The absorbance was read on a spectrophotometer at a wavelength of 540 nm. The data were normalized according to the formula:

(1) $$\begin{array}{c} \%\;\;\text{survival}=\frac{\text{DO}\;\;\text{sample}-\text{DO}\;\;\text{blank}}{\text{DO}\;\;\text{control}-\text{DO}\;\;\text{blank}}\times 100. \end{array}$$

The results were used to calculate the IC_50_ (50% inhibition of parasite growth). Amphotericin B was used as the reference drug. 

### 2.8. Activity against Intracellular Amastigotes

Peritoneal macrophages were cultured in 24-well plates (10^5^ cells/well), with coverslips, at 37°C and 5% CO_2_. The cells were infected with promastigotes forms of *L. amazonensis* using a ratio of 10 : 1 parasite/cell, and after 2 hours the cells were washed three times with PBS to remove free parasites. The infected cells were treated with different concentrations of VAEO and *α*-bisabolol (60–1.9 *μ*g/mL, for both products) in triplicate for 24 hours. The coverslips with the infected and treated cells were fixed with Bouin, stained with Giemsa, and examined by light microscopy. The inhibition percentage was calculated using the formula described by Guru et al. [25]: PI = 100 − (AN × 100)/(INA × TI), where NA is actual number of amastigotes/100 spleen cell nuclei after treatment; INA is initial number of amastigotes/100 spleen cell nuclei; TI is fold increase in the number of amastigotes in control animals on the corresponding day of the biopsy in treated animals; and PI is percentage inhibition. The IC_50_ was calculated with the GraphPad Prism. Amphotericin B was used as the reference drug. 

### 2.9. Cytotoxicity Assay

Macrophages J774.G8 were cultured in 96-well plates (5 × 10^5^ cells/mL) with different concentrations of VAEO and *α*-bisabolol (60–1.9 *μ*g/mL, both products) to a final volume of 200 *μ*L per well, at 37°C and 5% CO_2_. Wells without cells were used as blank, and wells with only cells were used as control. After 24 hours, the viability of the cells was evaluated by the tetrazolium-dye (MTT) colorimetric method modified by Mosmann [24]. MTT (5 mg/mL), a volume equal to 10% of the total, was added to each well. After 2 hours, the plate was centrifuged at 4000 rpm; then the supernatant was removed from each well and 100 *μ*L of DMSO was added to dissolve the formazan. The absorbance was read on a spectrophotometer at 540 nm wavelength. The data were normalized following the formula [24]:

(2) $$\begin{array}{c} \%\;\;\text{survival}=\;\frac{\text{DO}\;\;\text{sample}-\text{DO}\;\;\text{blank}}{\text{DO}\;\;\text{control}-\text{DO}\;\;\text{blank}}\times 100. \end{array}$$

The results were used to calculate the cell cytotoxicity by 50% (CC_50_) with GraphPad Prism 5.

### 2.10. Transmission Electron Microscopy

Promastigote forms of *L. amazonensis* were treated with VAEO and *α*-bisabolol at concentrations of 30, 15, 7.5, and 3.75 *μ*g/mL for 24 hours for both products. The parasites were fixed with 2.5% glutaraldehyde (Sigma, USA) in 0.1 M sodium cacodylate buffer, pH 7.2 overnight. Parasites were washed three times with 0.1 M sodium-cacodylate buffer and postfixed in a solution containing 1% osmium tetroxide, 0.8% ferrocyanide, and 5 mM calcium chloride, washed in 0.1 M sodium-cacodylate buffer, dehydrated in graded acetone, and embedded in epoxy resin. Ultrathin sections were stained with uranyl acetate and lead citrate and examined in a transmission electron microscope JEM-1011 (JEOL, Japan).

### 2.11. Statistical Analysis

The values were expressed as average ± S.D. The results were analyzed by Analysis of Variance (ANOVA) followed by the Tukey test. The analyses were performed with the software GraphPad Prism 5.0.4. Differences were considered significant at *P* < 0.05.

## 3. Results and Discussion

**Figure 1:**
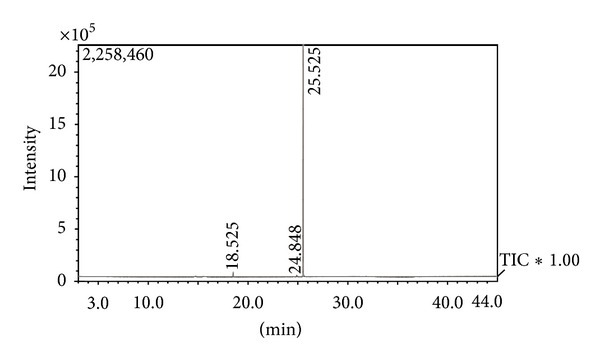
Chromatogram of the essential oil of *Vanillosmopsis arborea* Baker.

**Figure 2:**
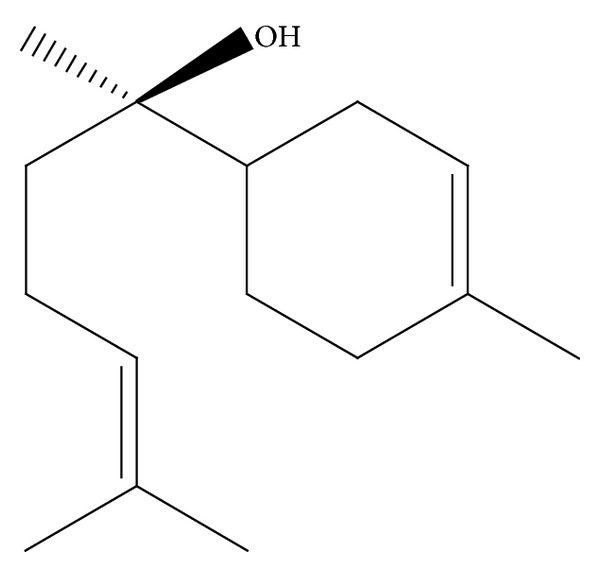
Chemical structure of *α*-bisabolol.

The chemical analysis revealed that *α*-bisabolol constituted 97.9% (25.5 min.) of VAEO (Figures 1 and 2). Other compounds identified were *o*-methyl eugenol (1.6%, 18.5 min.) and bisabolol oxide (0.5%, 24.9 min.). In another study by Santos et al. [26] the essential oil of *V. arborea* showed *α*-bisabolol as a major compound, however, at a different concentration (80.43%). The chemical characterization of the essential oil of *Vanillosmopsis pohlii*, too, identified *α*-bisabolol as a major compound at a concentration of 79.0% [27]. These differences may be due to seasonality and the time of collection of the plant sample [28].

**Figure 3:**
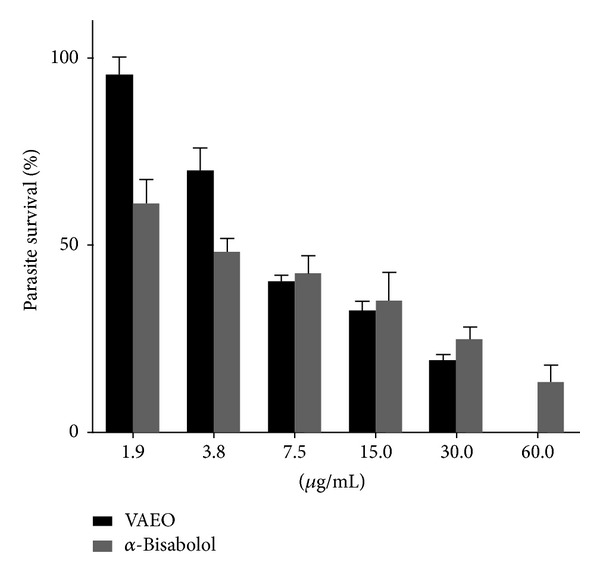
Effects of VAEO and *α*-bisabolol on *Leishmania amazonensis* promastigote forms. Each bar represents the mean ± standard deviation of three independent experiments in triplicate.

The incubation of VAEO and its major compound, *α*-bisabolol, efficiently inhibited the growth of *Leishmania amazonensis* promastigotes (Figure 3). The IC_50_/24 h values were 7.35 and 4.95 *μ*g/mL, respectively (Table 1). The *α*-bisabolol was more effective than thymol, the major compound of *Lippia sidoides*, which showed an IC_50_ of 22.63 *μ*g/mL against promastigotes of *L. amazonensis* [15]. In another study, *α*-bisabolol showed an IC_50_ of 10.99 *μ*g/mL against promastigotes of *Leishmania infantum*, which demonstrated its antiparasitic potential [29]. Despite having IC_50_ greater than *α*-bisabolol, VAEO showed significant inhibition against promastigotes of *Leishmania*, especially when compared to other plant species such as *Lippia sidoides* (IC_50_ 44.38 *μ*g/mL), *Cordia verbenacea*, *Cajanus cajan*, *Lantana camara* (IC_50_ 120, 62, and 14 *μ*g/mL, resp.) [30], and *Plectranthus amboinicus*, *Aristolochia cymbifera*, and *Lippia alba* (IC_50_ > 500 *μ*g/mL) [30]. 

**Figure 4:**
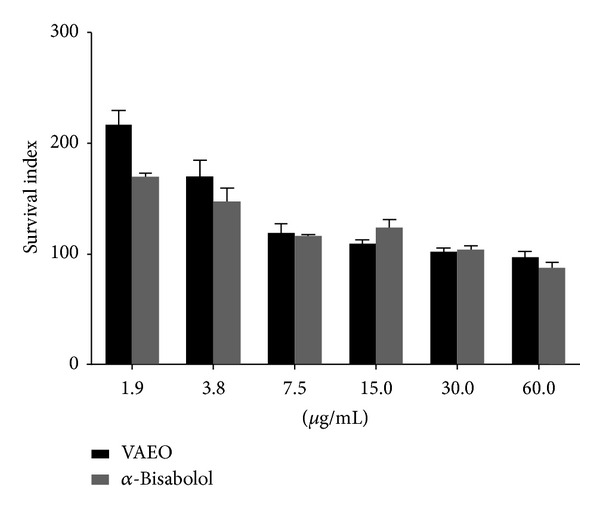
Effects of VAEO and *α*-bisabolol on *Leishmania amazonensis* intracellular amastigotes. Each bar represents the mean ± standard deviation of three independent experiments in triplicate.

Our results also show that, when the two products were compared, VAEO was less effective against promastigotes and intracellular amastigotes than *α*-bisabolol (Table 1 and Figure 4). The selectivity index (SI), especially for intracellular amastigotes, showed that *α*-bisabolol (9.383) was less toxic than VAEO (11.526). These values (9.383 and 11.526) were not statistically different, which confirms the role of the *α*-bisabolol in the leishmanicidal action of VAEO.

Both products tested showed no cytotoxicity against macrophages with CC_50_ 145 and 100.4 *μ*g/mL, respectively (Table 1), indicating no cytotoxicity, although the essential oils and their compounds have been reported to present cytotoxic effect when incorporated into the cell membrane [31]. The *α*-bisabolol is a sesquiterpene with low toxicity and is used in the cosmetic industry [32, 33] where it presents a pro-apoptotic activity [34]. Its leishmanicidal action against promastigotes and intracellular amastigotes may occur directly or indirectly through the production of cellular mechanisms, such as the production of nitric oxide (NO), which is the major effector molecule that participates in the intracellular killing of *Leishmania* [35]. The hydrophobic nature and the toxic effects of essential oils and their major constituents against various microorganisms are a common feature of plants which have volatile oils. These compounds can preferably incorporate in the cell membranes by inducing a loss of permeability to protons and ions and can induce changes considered vital for the cell; for example, the ergosterol biosynthetic pathway causes extensive lesions on the membranes of the *Candida* [31]. According to the results shown here, it is possible that *α*-bisabolol and VAEO have the ability to increase cell permeability to exogenous compounds, since some sesquiterpenes (particularly *α*-bisabolol) can induce changes in membranes allowing microorganisms to enter the cells and thus augmenting the microbial permeability to antimicrobial agents [36–38]. 

**Figure 5:**
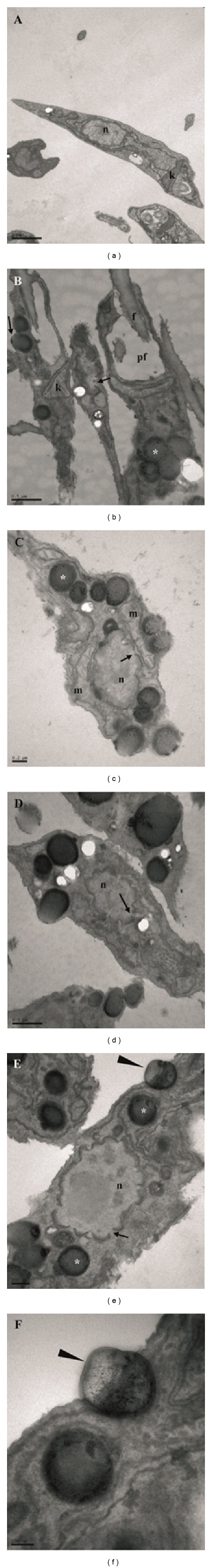
Transmission electron microscopy of *Leishmania amazonensis* promastigote with no treatment (a) or incubated with *α*-bisabolol (b and c) and VAEO (d, e, and f) at concentrations of 30 *μ*g/mL for both compounds. Also both treatments show the interaction of lipid droplets with swelling of the plasma membrane. **N**: nucleus; **k**: kinetoplast; **m**: mitochondria; **pf**: pocket flagellar. Bars 1.0 *μ*m (a); 0.5 *μ*m (b); 0.2 *μ*m (c); 1.0 *μ*m (d); 0.5 *μ*m (e); 200 nm (f).

The ultrastructural analysis of the promastigote control shows the parasite with an elongated cell body and the presence of a well-defined kinetoplast and nucleus (Figure 5).

The promastigotes treated with *α*-bisabolol for 24 hours, at a concentration of 30 *μ*g/mL (Figures 5(b) and 5(c)) presented severe cell damage with the loss of parasite morphology, discontinuity of the nuclear membrane, increased mitochondrial volume and kinetoplast, and presence of vesicles with an electrondense display (white asterisk—Figures 5(b) and 5(c)) with lipid inclusion in the plasma membrane (arrow—Figures 5(b) and 5(c)).

Many terpenes derived from essential oils, such as *α*-bisabolol, are bioactive, especially against different types of pathogens [39, 40]. Terpenes, hydrocarbons formed from units of isoprene, can easily penetrate the lipid bilayer of cellular membranes and can thus produce changes in the integrity of cell structures and mitochondrial membranes, for example [40].

The promastigotes treated for 24 h with VAEO at a concentration of 30 *μ*g/mL (Figures 5(d), 5(e) and 5(f)) showed increased volumes of flagellar pockets with consequent breakage, increased volumes and changes in mitochondrial kinetoplasts, abnormal condensation of chromatin in the nucleus (small arrow—Figure 5(f)), discontinuity of the nuclear membrane, lipid inclusions in the presence of electrondense vesicles (white asterisk—Figure 5(f)), and visualization of the inclusion of a lipid envelope within the plasma membrane (thin arrow—Figure 5(f)), with the consequent loss of parasite morphology. Similar structural changes have also been described in *Trypanosoma cruzi* [38] and other parasites treated with volatile compounds. Volatile compounds from plants have hydrophobic characteristics with excellent affinity for cell membranes of different microorganisms, including *Leishmania* parasites, which contribute significantly to the toxicity attributed to these compounds [29]. The lipid inclusions observed and the consequent accumulation of lipid precursors can result from the interference of the oil components, including *α*-bisabolol, on the lipid biosynthesis pathways, such as ergosterol [37], thus leading to morphological and structural changes affecting the survival of the parasite.

Our results suggest that the essential oil of *Vanillosmopsis arborea* and its major compound, *α*-bisabolol, show great antileishmanial potential, with the future possibility of a new alternative in the treatment for leishmaniasis or acting as an adjuvant antiprotozoan.

## 4. Conclusion

The results show that the essential oil *Vanillosmopsis arborea* exhibits leishmanicidal activity *in vitro* against *L. amazonensis* and that this activity is related to the presence of its major compound *α*-bisabolol. This major compound, *α*-bisabolol, represents over 97% of the composition of VAEO, and, when tested separately, it also showed similar leishmanicidal activity. The ultrastructural analysis showed that both products induced morphological changes with the presence of cytoplasmic lipid inclusions suggesting an action on the lipid metabolism of the parasite due to increased exocytic activity in the region of the flagellar pocket. Moreover, no cytotoxic effects were observed on the macrophages treated by either product. Further studies with the essential oil of *V. arborea* against cutaneous leishmaniasis will be carried out to demonstrate the potential of natural products derived from this medicinal plant species, thus contributing to advances in allopathic medicine as well as the development of techniques for the conservation of species with potential therapeutic use.

## Figures and Tables

**Table 1:** Leishmanicidal and cytotoxic activity of VAEO and its major constituent *α*-bisabolol.

Compounds	IC_50_ * µ*g/mL		CC_50_ * µ*g/mL	SI_ama_^a^
	Promastigote	Amastigote intracellular	J774.G8	
VAEO	7.35 ± 0.050	12.58 ± 0.068	145 ± 0.023	11.526
*α*-Bisabolol	4.95 ± 0.054	10.70 ± 0.085	100.4 ± 0.025	9.383
Amphotericin B	3.1 ± 0.048	7.8 ± 0.059	1.688 ± 0.4993	0.216

^a^SI_ama_ (selectivity index) = CC_50_ macrophage/IC_50_ amastigote.
